# Effects of Ashwagandha ( *Withania somnifera*) standardized root extract on physical endurance and VO _2max_ in healthy adults preforming resistance training: An eight-week, prospective, randomized, double-blind, placebo-controlled study

**DOI:** 10.12688/f1000research.130932.1

**Published:** 2023-03-24

**Authors:** Narsingh Verma, Sandeep Kumar Gupta, Sayali Patil, Shashank Tiwari, Ashok Kumar Mishra

**Affiliations:** 1 Physiology, King George’s Medical University, Lucknow, Uttar Pradesh, 226003, India; 2 Internal Medicine, M V Hospital and Research Centre, Lucknow, Uttar Pradesh, 226003, India; 3 Pharmacology, D. Y. Patil deemed to be University - School of Medicine, Navi Mumbai, Maharashtra, 400607, India; 4 Physiology, King George’s Medical University, Lucknow, Uttar Pradesh, 226003, India; 5 Clinical Research, M V Hospital and Research Centre, Lucknow, Uttar Pradesh, 226003, India

**Keywords:** Muscle strength, Cardiorespiratory endurance, Muscle girth, Resistance training

## Abstract

**Background**: Ashwagandha is a well-known Ayurvedic herb used for youthful vigor and wellbeing. This study investigated the effects of 300 mg standardized root extract (>5% withanolides) of Ashwagandha ( *Withania somnifera*) on muscle mass, strength and cardiorespiratory endurance following resistance training.

**Methods:** In this eight-week, parallel-group, multicenter, randomized, double-blind, placebo-controlled clinical study, 80 healthy male and female participants aged 18-45 years, who engaged in regular physical activity were randomly allocated in a 1:1 ratio to receive Ashwagandha (AG, n=40) 300 mg capsules twice daily for eight weeks, or identical placebo (PB, n=40). Seven (3 AG, 4 PB) participants were excluded due to poor compliance. All participants conducted eight-week resistance training. Study outcomes included muscle strength (1RM bench press and leg extension), muscle size (circumference of arm, chest and upper thigh) and cardio-respiratory endurance (VO _2max_) assessed at baseline and at eight weeks. Analysis of covariance (ANCOVA) was used to estimate adjusted differences based on sex, BMI and chest circumference at baseline.

**Results:** AG caused greater improvement in chest press (males: p = 0.0084; females: p = 0.0005), leg press (males: p = 0.0049; females: p = 0.018) and endurance (males: p <0.0001; females: p <0.0001) as compared to PB. Also, greater improvements in muscle girth for arm, chest and thigh were seen in both male and female participants with AG. No adverse events were reported in the study.

**Conclusions:** Eight weeks of AG root extract supplementation along with resistance training is effective in improving muscle strength, growth, endurance and recovery in both male and female participants. AG root extract could be a safer, effective and low-cost alternative for athletes to improve muscle endurance.

## Introduction

Resistance training is a specific conditioning technique in which an individual works against a broad spectrum of resistive loads to enhance general health, fitness and performance. ^1^ A resistance training program comprises a set of well-organized exercises and aids in rhythmic muscular contraction and relaxation against external resistance. The repetition of actions allows the human body to adapt to such resistance and induces strength and endurance over time. ^2^^–^^4^ In addition to strength development, resistance training improves immunity, bone and muscle health and overall function of all organs. ^5^ Furthermore, regular exercise alters biochemical and physiological processes. Biochemical assessments reveal that individuals who regularly participate in endurance exercise have significantly higher creatine kinase levels, which peak approximately 48 hours after the exercise. ^6^

A major component of physical fitness and resistance training is cardiorespiratory endurance. Maximal oxygen consumption (VO _2max_) refers to the maximum amount of oxygen that an individual can utilize during an intense exercise session and is considered a valid measure to estimate cardiovascular fitness. ^7^ Studies suggest that resistance training enhances VO _2max_ by improving heart-muscle function, increased blood volume and increased oxygen-carrying capacity. ^8^

Alongside resistance training and exercises, diet plays a key role in attaining physical and mental fitness and improving overall health. Traditionally, herbs and their extracts have been considered important dietary supplements with multiple health benefits. ^9^ Several studies have shown multifaceted efficacy of herbs as they can diminish recovery time, improve performance, enhance muscle mass and reduce fat levels. ^10^^,^^11^ This has resulted in a growing demand for safe and effective herbal supplements to improve endurance and strength performance among athletes and those with an active lifestyle. ^12^ *Withania somnifera*, popularly known as Ashwagandha (AG), is a plant that belongs to the Solanaceae family and grows in arid or semi-arid regions. AG is one of the most frequently used medicinal plants in the Ayurvedic system of complementary medicine for a varied range of ailments. It has been used to treat musculoskeletal conditions and improve general vitality and quality of life. ^13^^–^^15^ Pharmacologically, AG has been explored extensively for its potential as a versatile health supplement. ^13^

Sandhu *et al.* (2010) studied the impact of 500 mg of AG supplementation for eight weeks on aerobic exercise performance and the activity of associated muscles in healthy young adults. There was a significant increase in VO _2max_ and muscular power. ^16^ In another study, Raut *et al.* (2012) explored the safety, tolerability and activity of 750 mg to 1250 mg of AG in healthy volunteers for 30 days and found that AG supplementation increased muscle strength, improved sleep quality and reduced lipid concentrations. ^17^ Wankhede *et al.* (2012) also demonstrated that as in adjunction to a resistance training program, 600 mg of AG supplementation for eight weeks increased muscle strength in untrained male adults. ^18^ Looking at these previous studies, the current study was based on a hypothesis that AG will lead to a significant improvement in muscle strength, muscle growth and cardio-respiratory endurance in the study participants.

Although preliminary studies mentioned above have been conducted, robust human clinical trials evaluating the performance-enhancing effects of standardized AG root extract are still limited. A previously published study evaluating muscle strength focused only on male participants. ^18^ Physical endurance is considered higher in males owing it to the testosterone hormone. Therefore, this randomized, double-blind, placebo-controlled study aims to examine the effects of a standardized AG root extract (as a complement to a resistance training program) on changes in muscle strength, muscle growth and cardio-respiratory endurance in active, healthy participants of either sex.

## Methods

### Ethical approval

The study was approved by the Institutional Ethics Committees of both King George Medical University (Reference number. #202/IEC/R.Cell-18) and M. V. Hospital and Research (Reference number #IEC/01/28/18) and it was prospectively registered with the Clinical Trials Registry of India (Registration# CTRI/2018/07/014969).

### Study design

This was a parallel-group=eight-week, multicenter, randomized, double-blind, PB-controlled study. The study was approved by respective Institutional Ethics Committees (IECs) from both study sites – King George Medical University (Reference no. #202/IEC/R.Cell-18) and M. V. Hospital and Research (Reference no. #IEC/01/28/18). It was also prospectively registered with the Clinical Trial Registry of India (Registration #CTRI/2018/07/014969; on 19/07/2018). In addition, it was conducted in compliance with the Declaration of Helsinki guidelines, the New Drugs and Clinical trials rules 2019 and the Indian Council of Medical Research (ICMR) ethical guidelines for biomedical research on human subjects.

### Recruitment and randomization

The recruitment of participants was performed by circulating printed fliers in the purlieu of a gymnasium which served as the site for the training program. This brochure was approved by both institutional ethics committees. Recruitment of participants started on 11 September, 2018 (first visit of first participant) and ended on 28 March, 2019 (last visit of last participant). Subjects were randomly and equally allocated to two groups in 1:1 ratio using stratified randomization (male and female) to receive either an AG capsule standardized root extract (n=40), or PB which looked identical to AG capsule (n=40). The randomization was computer-generated through randomly permuted blocks of 20. Within each block, the number of participants assigned to each of the two treatment arms was equal. The AG and PB capsules were manufactured and packed in identical containers and labeled equivalently, along with unique serial numbers to ensure blinding. The study centers received numbered and sealed envelopes that contained no information about treatment allocation.

### Subjects

All participants read and signed an informed consent form before final selection and enrollment in this study. Healthy adults of either sex aged between 18 to 45 years engaging in regular physical activity (gymnasium/strength training exercise at least three months before screening for this study) were considered eligible. Sex of the participants was defined based on self-reporting. Participants agreed to engage in the same exercise schedule and diet regime for the study period as prescribed in the study protocol and if of child-bearing age, agreed to use barrier birth control measures. Participants were excluded if they were taking any nutritional supplements, medications, or steroids used to enhance physical performance. Those with a history of drug abuse, who smoked more than 10 cigarettes a day, or were habitually consuming more than 14 grams of alcohol per day were also excluded. Other exclusion criteria were: any planned participation in any sporting event during the study period; a weight loss of >5 kg in the previous three months; a history of any orthopedic injury or surgery in the past six months; any known hypersensitivity to AG; participation in any clinical study in the past three months; a history of heart disease, diabetes, depression, stroke or neurological disorder. Participants were also excluded if they were taking anti-hypertensive drugs, beta-blockers, beta-agonists, hormonal contraceptives, corticosteroids, or psychotropic substances over the previous three months.

### Interventions

Capsules containing either 300 mg of AG standardized root extract (KSM-66, Ixoreal Biomed, CA, US) or 300 mg starch (Shri Kartikeya Pharma, Hyderabad, India) were used for AG and PB groups respectively. Both the AG and PB formulations were cellulose-based hard capsules and identical in appearance, weight, texture and color. Participants were required to take one capsule twice daily with milk or water.

The AG root extract is a light yellowish powder extracted using a proprietary process. The green extraction process is devoid of any alcohol or similar solvents, hence maintaining the standards as recommended by traditional Ayurveda. The product, KSM-66 Ashwagandha, contains a high concentration of root extract of this herb and more than 5% of total withanolides as standardized by high-performance liquid chromatography. The batch number of product used was KSM/VG/18/1020. The chemical profile of the study product was confirmed by Shri Kartikeya Pharma, Hyderabad, India.

### Sample size

The target sample size was estimated using G*Power (Version 3.1.9.3). It was based on a previously published randomized controlled clinical study evaluating the effect of an AG root extract on muscle strength and recovery. ^18^ A recently published systematic review on AG also supported this kind of improvement (effect size of 0.67) in physical performance. ^19^ Considering the previous study, we hypothesized that AG treatment is better than PB by an effect size of 0.6 with regards to change in the one-repetition maximum (1 RM) chest press exercise after eight weeks. To detect an effect size of 0.6 in a two-parallel-group design (1:1) using independent Student t-test, with a 5% risk of type 1 error (alpha) and 80% power, 40 subjects per group were required while considering a 10% drop-out rate. Thus, 80 healthy male and female subjects were recruited in the study.

### Resistance training exercise program

**Figure 1.**
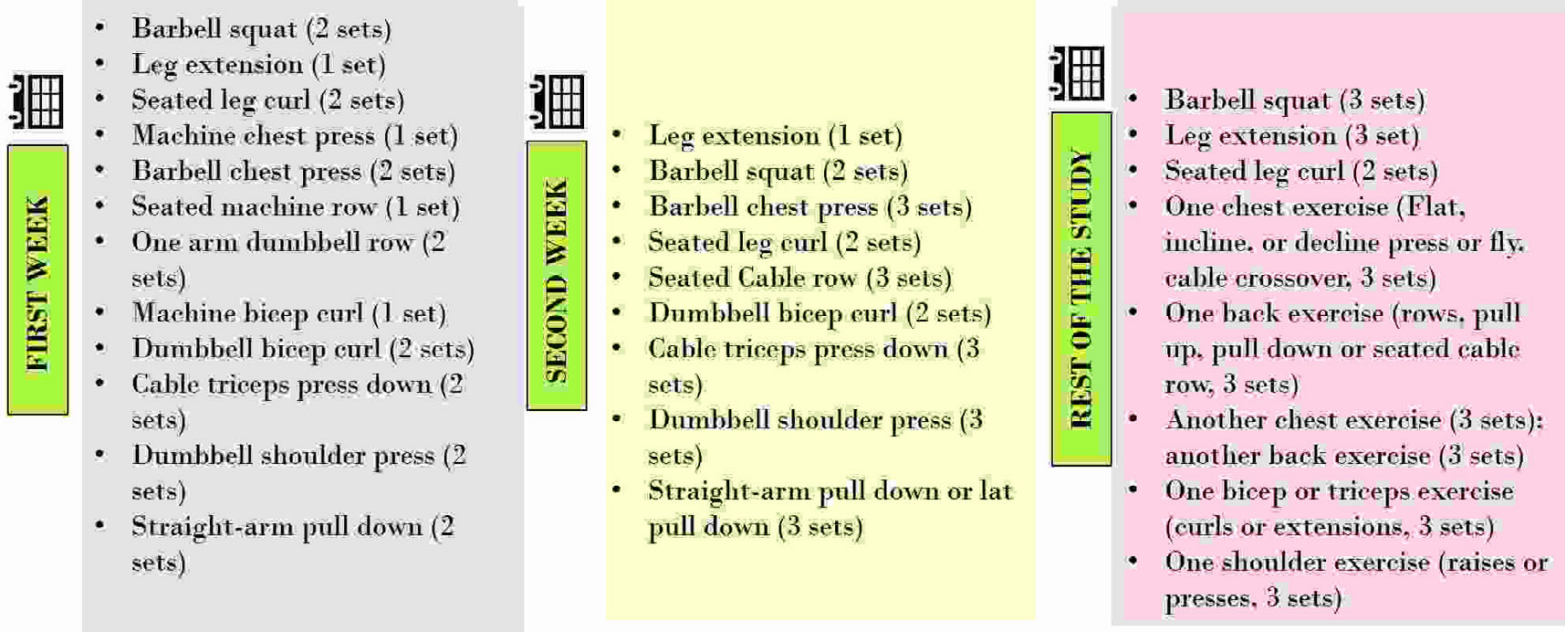
Exercise protocol.

The resistance training regimen used in this study was focused on improving muscle strength, increasing muscle mass and enhancing cardiorespiratory endurance. Various resistance exercises were chosen with the objective of training targeted muscle groups. Such training programs target the upper and lower body and consist of a series of exercises with suitable resistance, grouped into multiple sets and repetitions as per the National Strength and Conditioning Association’s (NSCA) regulations and guidelines. ^20^ Each participant was required to complete the training session every alternate day with a one-day complete rest per week. Therefore, participants were training three days per week. Each training session started with a warm-up of low intensity aerobic exercise. Participants were instructed to perform the maximum repetitions possible for each set until exhaustion. The exercise program is detailed in Figure 1, comprising exercises for week 1, week 2 and remainder of the study (weeks 3 to 8).

Data for this study is available in the online repository ‘Figshare’ under the dataset named ‘Ashwagandha_Muscle study’ ( *Underlying data*). ^34^

### Study outcomes

**Primary outcome measures**

***Muscle strength***

Changes in upper and lower body muscular strength were the primary efficacy assessment of this study. As per common practice in sports medicine, muscle strength was assessed by one-repetition maximum (1RM) for bench press and leg press. 1RM testing was performed using NSCA’s protocol. ^20^ 1 RM refers to the maximal load lifted by a participant for one cycle of the exercise. Muscle strength measurements were conducted at baseline (first day of training) and end of the study (after the completion of eight weeks of training and supplementation). The equipment used was a Bench Press (SL7028) manufactured by Impulse and a Leg extension (GCEC340) manufactured by Body Solid Inc.

**Secondary outcome measures**

***Muscle girth***

Muscle size was measured at three sites - arm (flexed mid-upper arm), chest (sternum at mid-tidal volume) and upper thigh (just inferior to gluteal fold) of each participant. Measurements were undertaken on the first day of training (baseline) and at end of the study (day 56). Maximal cross-sectional area (CSA) was measured for thigh and arm using the Moritani-DeVries method, ^21^ whereas, for chest measurement, girth was measured at the level of middle of the sternum by passing a measuring tape under both arms at the end of normal expiration.

***Cardiorespiratory endurance (maximum rate of oxygen consumption)***

Resistance training and endurance are highly correlated. ^22^^,^^23^ Cardiorespiratory endurance measures overall body performance during high-intensity exercises. Studies indicate that resistance training induces an increase in the maximal rate of oxygen consumption (VO _2max_). ^22^^,^^23^ VO _2max_ is measured during incremental exercise, most typically on a motorized treadmill. Bruce protocol was used to assess the treadmill test outcomes of all participants. Test score was considered as time taken for the test in minutes and it was converted to estimate a VO _2max_ score as per the Bruce protocol. ^22^

***Safety assessment and adverse events***

Clinical safety was assessed based on the frequency of adverse events reported by the participants. In addition to this subjective report, standard biochemistry tests were also performed along with measurement of vital signs.

### Statistical analysis

Data was entered in Microsoft Excel spreadsheet using manual double-entry method to ensure data accuracy. Statistical analyses were done using MedCalc (version 20.011). Efficacy analysis was done on the modified intention to treat (ITT) dataset (n=73), and safety analysis was done on the whole dataset (n=80). Data for continuous variables are presented as means with standard deviation (SD), whereas categorical and discrete data are presented as counts with percentages. Unpaired t-test was used to assess between-group differences. Change in values form baseline to eight weeks were computed from and compared between the two groups using unpaired t-test. Effects of sex, BMI and chest circumference on different parameters were analyzed using analysis of covariance (ANCOVA). Adjusted means with 95% confidence intervals (CI) and effect size (Cohen’s d) were presented for change from baseline. Criteria for effect sizes were based on the standard criteria (<0.2, trivial; 0.2–0.6, small; 0.6–1.2, moderate; 1.2–2.0, large; 2.0–4.0, very large; and >4.0, nearly perfect). ^24^

Normality assumptions were checked on all variables using a one-sample Shapiro-Wilk test. A p-value lower than 0.05 was considered the threshold to claim statistical significance.

## Results

### Participant demographics

**Figure 2.**
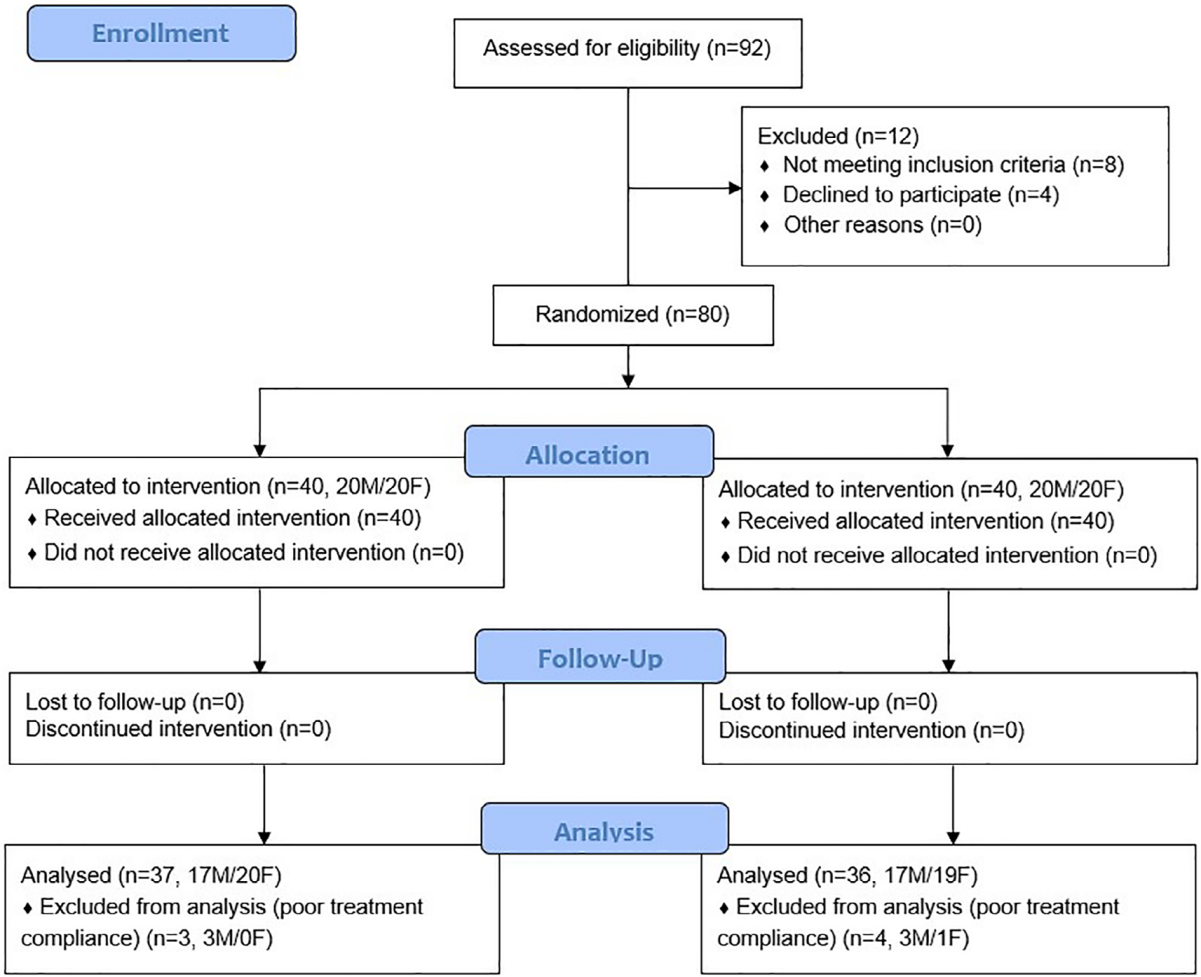
CONSORT flow chart.

A total of 92 people were screened for participation in the study, of which 80 met the inclusion criteria and were enrolled in the study ( Figure 2). All enrolled participants completed the eight-week follow-up. However, four subjects (3 males and 1 female) in the PB and three subjects (3 males) in the AG group did not consume supplementation after their second visit. These participants were documented as having poor compliance to treatment and were excluded from the efficacy analysis. Reasons for premature medication discontinuation were reported as unintentional forgetfulness due to travel and other personal issues by these seven subjects. Baseline demographic characteristics and vital signs are detailed in Table 1 and indicate that the study population was homogenous with no significant differences between the treatment and control groups. Baseline parameters across both the groups are displayed in Table 2.

**Table 1.** Baseline demography and vital parameters in two groups.

		AG (n=37)	PB (n=36)	Unpaired t-test
		M=17/F=20	M=17/F=19	
		Mean (SD)	Mean (SD)	p
Age (yrs.)	Male	25.18 (4.38)	23.35 (3.87)	0.165
	Female	25.25 (4.49)	23.95 (4.79)	0.528
	Total	25.22 (4.38)	23.67 (4.33)	0.155
Pulse (per min)	Male	78.41 (3.37)	77.65 (5.13)	0.484
	Female	79.05 (4.52)	76.95 (2.12)	0.264
	Total	78.76 (4.00)	77.28 (3.81)	0.213
Systolic BP (mm Hg)	Male	126.76 (5.95)	124.59 (4.49)	0.589
	Female	126.00 (5.24)	127.11 (5.54)	0.476
	Total	126.35 (5.51)	125.92 (5.16)	0.321
Diastolic BP (mm Hg)	Male	79.18 (3.52)	78.18 (4.30)	0.936
	Female	78.30 (4.59)	79.63 (4.37)	0.439
	Total	78.70 (4.10)	78.94 (4.34)	0.165

AG: Ashwagandha; PB: Placebo.

**Table 2.** Baseline parameters in two groups.

		AG (n=37)	PB (n=36)	Unpaired t-test
		M=17/F=20	M=17/F=19	
		Mean (SD)	Mean (SD)	p
Weight (kg)	Male	62.07 (6.66)	60.29 (4.73)	0.351
	Female	54.90 (3.96)	54.33 (4.28)	0.706
	Total	58.19 (6.41)	57.14 (5.36)	0.451
BMI (kg/sq.m)	Male	23.52 (1.05)	22.48 (1.87)	0.059
	Female	23.02 (0.94)	22.73 (1.43)	0.382
	Total	23.25 (1.01)	22.62 (1.63)	0.049
Body fat (%)	Male	23.82 (2.70)	25.03 (2.26)	0.157
	Female	28.96 (5.07)	28.27 (3.38)	0.771
	Total	26.60 (4.85)	26.74 (3.30)	0.718
1-RM Chest Press (kg)	Male	51.83 (7.82)	54.85 (7.53)	0.261
	Female	49.09 (5.00)	51.37 (6.37)	0.221
	Total	50.35 (6.50)	53.01 (7.06)	0.270
1-RM Leg Press (kg)	Male	60.06 (6.37)	59.52 (9.46)	0.848
	Female	59.98 (6.66)	59.70 (8.44)	0.907
	Total	60.02 (6.44)	59.62 (8.80)	0.553
Max. VO _2_ (ml/kg/min)	Male	38.24 (1.44)	37.82 (2.40)	0.804
	Female	28.80 (1.36)	28.74 (1.41)	0.817
	Total	33.14 (4.96)	33.03 (4.98)	0.982
Mid-arm circumference (cm)	Male	31.06 (2.95)	30.18 (2.10)	0.185
	Female	29.25 (3.04)	27.26 (3.09)	0.063
	Total	30.08 (3.09)	28.64 (3.02)	0.029
Thigh circumference (cm)	Male	58.47 (4.21)	55.82 (10.01)	0.307
	Female	57.76 (6.11)	57.29 (7.42)	0.894
	Total	58.09 (5.26)	56.60 (8.64)	0.396
Chest circumference (cm)	Male	92.24 (6.00)	88.24 (7.54)	0.019
	Female	88.20 (3.55)	84.84 (4.21)	0.031
	Total	90.05 (5.17)	86.44 (6.17)	0.002

AG: Ashwagandha; PB: Placebo.

### Muscle strength

Changes in 1 RM bench press and leg press across both treatment groups throughout the eight-week trial period are detailed in Table 3. There were significant differences between the groups in both 1 RM bench press (males: effect size, 0.91; p=0.0084; females: effect size, 1.56; p=0.0005; total: effect size, 1.14; p<0.0001) and 1 RM Leg press (males: effect size, 1.22; p=0.0049; females: effect size, 0.63; p=0.018; total: effect size, 1.11; p=0.0005). A within-group analysis in AG group demonstrated a significant (p<0.0001) increase of 23.5 % and 22.8% in 1 RM bench press and an increase of 15% and 9.9% in 1 RM leg press over time and in both male and female participants respectively. Table 4 presents the comparison of AG and PB for change (unadjusted and adjusted for baseline values) in 1 RM leg press and 1 RM bench press from baseline to eight weeks.

**Table 3.** Change from baseline at 8 weeks in two groups (Univariate tests).

		AG (n=37)	PB (n=36)	Unpaired t-test
		M=17/F=20	M=17/F=19	
		Mean (SD)	Mean (SD)	‘p’
Weight (kg)	Male	1.375 (1.141)	1.180 (1.224)	0.605
	Female	1.210 (0.893)	0.780 (0.786)	0.114
	Total	1.293 (1.015)	0.980 (1.035)	0.177
BMI (kg/sq.m)	Male	0.525 (0.460)	0.455 (0.467)	0.636
	Female	0.520 (0.391)	0.315 (0.323)	0.079
	Total	0.523 (0.422)	0.385 (0.403)	0.140
Body fat (%)	Male	-1.640 (0.338)	-1.725 (0.311)	0.413
	Female	-1.740 (0.233)	-1.755 (0.231)	0.839
	Total	-1.690 (0.291)	-1.740 (0.271)	0.428
1RM Chest Press (kg)	Male	10.745 (8.532)	8.775 (7.387)	0.440
	Female	11.230 (6.112)	9.025 (5.182)	0.226
	Total	10.988 (7.330)	8.900 (6.299)	0.176
1RM Leg Press (kg)	Male	8.030 (7.419)	6.160 (5.873)	0.382
	Female	5.935 (10.496)	2.750 (9.891)	0.330
	Total	6.983 (9.034)	4.455 (8.213)	0.194
Max. VO _2_ (ml/kg/min)	Male	3.605 (1.629)	1.420 (0.529)	<0.0001
	Female	2.075 (0.922)	0.960 (0.575)	<0.0001
	Total	2.840 (1.519)	1.190 (0.593)	<0.0001
Mid-arm circumference (cm)	Male	1.590 (0.408)	1.250 (0.366)	0.009
	Female	1.490 (0.670)	1.240 (0.614)	0.226
	Total	1.540 (0.549)	1.245 (0.499)	0.014
Thigh circumference (cm)	Male	3.405 (3.302)	2.925 (1.084)	0.540
	Female	0.780 (0.089)	0.645 (0.201)	0.009
	Total	2.093 (2.661)	1.785 (1.388)	0.519
Chest circumference (cm)	Male	3.770 (1.711)	2.900 (1.539)	0.099
	Female	3.520 (2.151)	2.020 (0.797)	0.006
	Total	3.645 (1.922)	2.460 (1.289)	0.002

Within group changes (baseline versus week 8) significant at p<0.05 (paired t-test); AG: Ashwagandha; PB: Placebo.

**Table 4.** Comparison between AG and PB for change from baseline at 8 weeks in two groups.

	Difference (AG – PB)		Effect size			Power
	Unadjusted Mean (95% C.I.)	Adjusted Mean (95% C.I.)	Unadjusted	Adjusted	‘p’	
Weight (kg)	0.450 (-0.01 to 0.91)	0.706 (0.22 to 1.19)	0.324	0.483	0.005	0.695
BMI (kg/sq.m)	0.194 (0.01 to 0.38)	0.296 (0.10 to 0.49)	0.343	0.497	0.004	0.733
1RM Chest Press (kg)	2.062 (-1.08 to 5.20)	2.538 (-0.96 to 6.04)	0.218	0.241	0.152	0.214
1RM Leg Press (kg)	2.674 (-1.52 to 6.87)	4.417 (-0.02 to 8.85)	0.212	0.332	0.051	0.418
Max.VO _2_ (ml/kg/min)	1.544 (0.99 to 2.10)	1.591 (1.08 to 2.10)	0.930	1.034	<0.0001	1.000
Body fat (%)	0.070 (-0.04 to 0.18)	0.073 (-0.05 to 0.20)	0.209	0.193	0.251	0.238
Mid-arm circumference (cm)	0.324 (0.07 to 0.58)	0.430 (0.16 to 0.70)	0.420	0.526	0.002	0.865
Thigh circumference (cm)	0.301 (-0.73 to 1.34)	0.228 (-0.65 to 1.11)	0.097	0.086	0.607	0.063
Chest circumference (cm)	1.273 (0.49 to 2.06)	1.336 (0.59 to 2.08)	0.538	0.596	0.001	0.910

Adjusted values based on Analysis of Covariance for change from baseline (Covariates appearing in the model are evaluated at the following values: Sex=1.50, baseline BMI=22.83 kg/sq.m, baseline chest circumference=88.18 cm); AG: Ashwagandha; PB: Placebo.

### Muscle size

Changes in muscle size across the two treatment groups during the study period are detailed in Table 3. At the end of the study, there was a significant increase in both chest circumference (Total participants: p=0.019) and arm circumference (Total participants: p<0.0001), among the participants in AG group as compared to the PB group. However, no improvement was seen in thigh circumference when compared to PB group. A within-group analysis in AG group demonstrated a significant increase in the chest circumference (males: p<0.0001; females: p<0.0001), mid-arm circumference (males: p<0.0001; females: p<0.0001) and thigh circumference (males: p=0.0003; females: p<0.0001). Table 4 presents the comparison of AG and PB for change (unadjusted and adjusted for baseline values) for mid-arm, thigh and chest circumference from baseline to eight weeks.

### Endurance

Changes in the VO _2max_ across the two treatment groups throughout the eight-week trial period are detailed in Table 3. At the end of the study, there was a statistically significant between-group difference in VO _2max_ values (males: p<0.0001; females: p=0.0001), when compared with the PB group. In the AG group=a statistically significant 9.5% and 7% increase were observed over time in both males (p<0.0001) and females (p<0.0001), respectively. Table 4 presents the comparison of AG and PB for change (unadjusted and adjusted for baseline values) in VO _2max_ from baseline to eight weeks. The analysis for VO _2max_ yielded 100% power and greater changes were observed with AG as compared to placebo (effect size, 1.034, p<0.0001).

**Figure 3.**
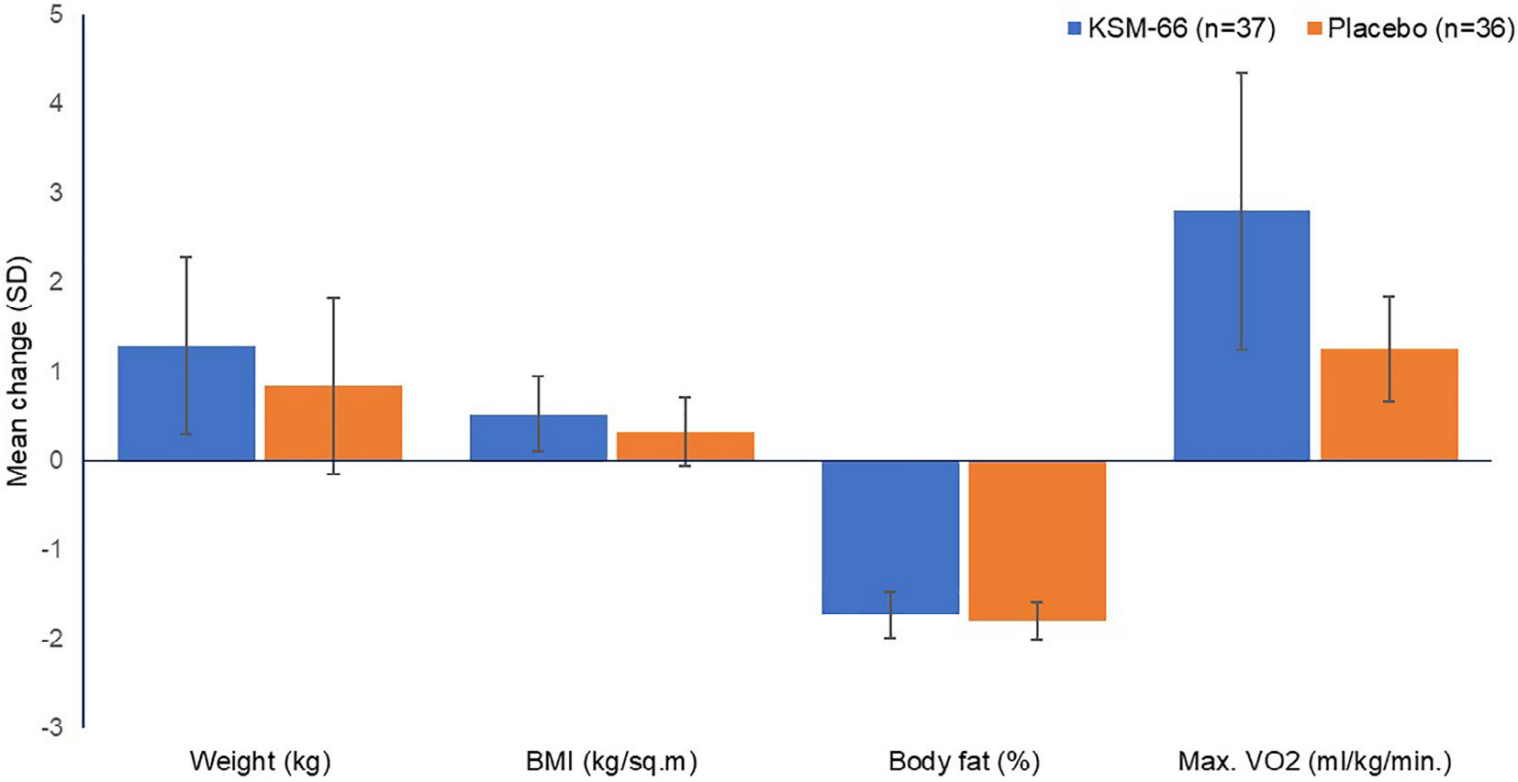
Mean (SD) change from baseline in body anthropometry and VO _2max_.

**Figure 4.**
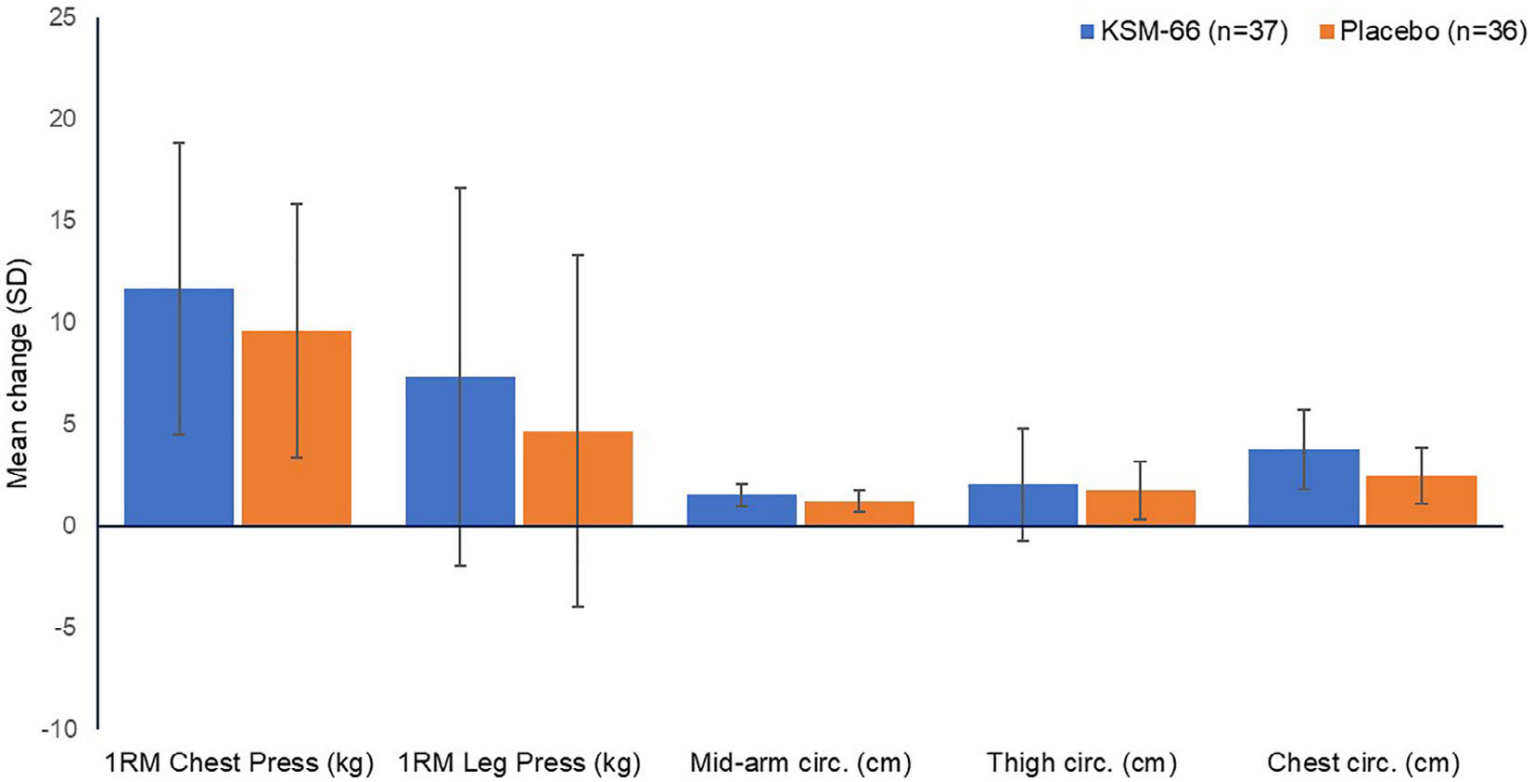
Mean (SD) change from baseline in physical endurance parameters.

Figures 3 and 4 depict changes across all the parameters between the two study groups.

### Adverse events and safety parameters

The participants did not report any adverse events during the study period. Additionally, we conducted an array of routine clinical tests such as hematology, renal function, liver function and thyroid function tests to evaluate the safety and tolerability of AG root extract supplementation in participants. Physical examination and vital signs were collected as well. At the screening visit, all tested parameters were within the normal ranges. None of the parameters in the study samples showed any abnormal changes at the end of the intervention.

## Discussion

The present study assessed the impact of AG root extract supplementation on resistance training adaptations such as muscle strength and endurance in healthy and active adults. The primary findings of the study are that significantly greater improvements in muscle strength for both lower body (1 RM Leg press) and upper body (1 RM Chest press) occurred in participants consuming AG as compared to PB. In addition, AG supplementation significantly increased muscle size and endurance. However, there were no differences in thigh muscle size (male) and body composition (body fat) between the two groups.

The impact of resistance exercise training on increasing muscular strength was better (p <0.05) with AG (23%) versus PB (8%). Similarly, a previous study by Wankhede *et al.* (2012) reported significant improvements in both bench press and leg extension after eight weeks of the AG supplement in healthy adult males. ^18^ However, these improvements were greater in comparison to the current study, which could be due to the fact that experiment was conducted on inexperienced individuals in the previous study versus resistance-trained adults in the current investigation. Earlier studies recommended that along with strength and muscle growth, factors such as human growth hormone and testosterone were found higher in males compared to females. ^25^ Our study also measured the muscular strength of male and female participants (1 RM bench press and leg press), and a significantly greater outcome was found in males than female participants with an exception of 1 RM bench press in females, both before and after the resistance training program. Furthermore, due to resistance training, general development from the baseline parameters was observed for all the participants. This suggests that the improvement was of clinical relevance (effect size, 0.9, indicating larger effect) which is an improvement over the meta-analysis conducted by Bonilla *et al.* (2021) ^19^ that analyzed twelve clinical trials of AG on physical performance; however, only two trials were relevant for muscular strength.

The percentage of adults whose chest, thigh and mid-arm muscle size increased differed significantly between the two study groups, although men’s thigh size remained similar in both groups. Furthermore, endurance measured by VO _2max_ also increased significantly in AG group which suggests that the effect, although small in magnitude (8%), might be clinically relevant. This observation is lower compared to a previous study by Tiwari *et al.*, ^26^ which reported a 16% improvement in VO _2max_. Similarly, another study by Choudhary *et al.* ^27^ observed 13% increase in VO _2max_ after eight weeks of AG supplementation. Therefore, the current improvement is comparable and consistent with literature review.

No significant benefit was seen with respect to body fat ( Figure 3), as assessed by the body composition monitor. Slight improvement was seen in AG group, but there was no significant difference between the two groups. However, the study was not powered enough to assess the change in body fat.

AG was well tolerated by the subjects and no serious side effects were reported. This finding is consistent with previously published studies on AG ( *Withania somnifera*) root extract in healthy volunteers, which also advocated the herb’s tolerance. ^17^^,^^18^^,^^28^^,^^29^ Thus, it could be a safer alternative to improve and maintain physical performance.

Increased levels of anabolic hormone (serum testosterone) may be the cause of improved muscular strength, which could be associated with its structural similarities to withanolides (major constituents of AG root extract). ^30^ Most of the studies included in the meta-analysis by Bonilla *et al.*, which involved muscle strength, endurance and recovery might be owed to the antioxidant properties of this plant. ^19^ In addition, as previously shown, AG alleviates stress, improves sleep and physical performance, ^14^^,^^31^^–^^33^ even though these were not measured in this study.

Our trial had certain strengths, including enrollment of resistance-trained adult men and women, adequate sample size, a double-blind, PB-controlled design, excellent participant retention and use of standardized AG extract. However, one noteworthy drawback was the lack of quantitative data on the subjects’ food intake. They were, however, instructed not to change and follow the specified diet for entire length of the trial. Although the subjects claimed to have followed the instructions (as per subject’s diary), we were unable to determine if these factors influenced the effect of AG due to a lack of reliable detailed quantitative information on daily calorie and protein intake. Another limitation is that our research focused on resistance-trained adults for eight weeks, so extrapolating results to other groups for a longer duration should be done with caution. As a result, in future studies, we advocate a comprehensive dietary analysis of participants' daily calorie and protein consumption, as well as a longer-term follow-up with diverse populations.

## Conclusions

In conclusion, eight weeks of AG root extract supplementation in combination with resistance training is effective in promoting muscle strength, muscle growth and can also improve endurance in both male and female participants. The study also indicated that the participants have tolerated the AG root extract well. Thus, AG root extract could probably represent a safer low-cost alternative for athletes.

## Author contributions

All the authors of this study contributed to the project equally. NV supervised the study at the KGMU hospital and led the study. SKG supervised the study at MV hospital. SP worked on review and editing of the article. ST helped in data collection and analysis. AKM conceptualized the study, helped in data analysis and writing the manuscript. All the authors participated in study designing and implementation and all the authors have gone through the final article and approved it for submission. Every author actively participated in drafting the manuscript and approved the final version of the article.

## Data Availability

Figshare: Ashwagandha_Muscle study, https://doi.org/10.6084/m9.figshare.22081895.v3. ^34^ The project contains the following underlying data:
- Ashwagandha_Muscle study_Raw data.csv - Ashwagandha_Muscle study_Raw data.xlsx - Filled consort checklist - Study protocol Figshare: CONSORT checklist for ‘Effects of Ashwagandha ( *Withania somnifera*) standardized root extract on physical endurance and VO _2max_ in healthy adults preforming resistance training: An eight-week, prospective, randomized, double-blind, placebo-controlled study’. https://doi.org/10.6084/m9.figshare.22081895.v3. ^34^ Data are available under the terms of the Creative Commons Zero “No rights reserved” data waiver (CC0 1.0 Public domain dedication).
