# Supplementary material for: Effects of Ashwagandha ( Withania somnifera) standardized root extract on physical endurance and VO 2max in healthy adults performing resistance training: An eight-week, prospective, randomized, double-blind, placebo-controlled study
Source: F1000Res. 2024 Apr 8;12:335. Originally published 2023 Mar 24. [Version 2] doi: 10.12688/f1000research.130932.2 (PMC11234080; doi:10.12688/f1000research.130932.2)
Supplement: Peer review history

# Reviewer report: Reviewer response for version 2

**DOI:** 10.5256/f1000research.163630.r287564

**Published:** 2024-07-09

**Authors:** Giuseppe Potrick Stefani

**Affiliations:** 1 Pontifícia Universidade Católica do Rio Grande do Sul (PUCRS), Porto Alegre, Brazil

This work is a randomized, double-blind, placebo-controlled clinical trial, in which researchers evaluated the effect of Withania somnifera supplementation on different markers of body composition and maximum strength. The writing of the manuscript is adequate, although it presents some necessary changes to improve the reading agreement. The present study advances the knowledge about phytochemicals, especially about Withania somnifera. However, it presents some limitations that should be highlighted to readers. Likewise, he left some questions with the aim of better understanding the experiment that the authors conducted:

 • Inform methodology for evaluating the percentage of body fat;

 • Is the evaluator of body composition parameters an ISAK-certified anthropometrist?

 • What is the technical intra-evaluator measurement error for perimeters and skinfolds?

 • How was the participants’ supplementation controlled? This must be described in the methodology and results of adhesion to the capsules.

 • Did the method for assessing the perimeter of the lower and upper limbs include only the total perimeter or corrected by skinfold thickness? The entire experimental procedure must be described if it was the second method.

 • Did the authors analyse food consumption close to the strength and VO2max tests? The absence of these data is a limiting factor for data interpretation and should be highlighted to readers if quantification control has not been carried out.

Is the work clearly and accurately presented and does it cite the current literature?

Yes

If applicable, is the statistical analysis and its interpretation appropriate?

Yes

Are all the source data underlying the results available to ensure full reproducibility?

Yes

Is the study design appropriate and is the work technically sound?

Yes

Are the conclusions drawn adequately supported by the results?

Partly

Are sufficient details of methods and analysis provided to allow replication by others?

Partly

Reviewer Expertise:

Sports Nutrition, Physiology, Metabolism, Body Composition, Dietary Supplements.

I confirm that I have read this submission and believe that I have an appropriate level of expertise to confirm that it is of an acceptable scientific standard, however I have significant reservations, as outlined above.

# Reviewer report: Reviewer response for version 2

**DOI:** 10.5256/f1000research.163630.r264045

**Published:** 2024-04-22

**Authors:** Mark E. T. Willems

**Affiliations:** 1 Institute of Applied Sciences, College Lane, University of Chichester, Chichester, England, UK

Thanks for addressing all my comments.

 I have no further essential comments. The authors may want to change the units for VO2max to "mL / (kg min)" or mL x kg ^-1^ x min ^-1^.

Is the work clearly and accurately presented and does it cite the current literature?

Yes

If applicable, is the statistical analysis and its interpretation appropriate?

Yes

Are all the source data underlying the results available to ensure full reproducibility?

Yes

Is the study design appropriate and is the work technically sound?

No

Are the conclusions drawn adequately supported by the results?

No

Are sufficient details of methods and analysis provided to allow replication by others?

No

Reviewer Expertise:

Exercise Physiology, Training, Sports Nutrition

I confirm that I have read this submission and believe that I have an appropriate level of expertise to confirm that it is of an acceptable scientific standard.

# Reviewer report: Reviewer response for version 1

**DOI:** 10.5256/f1000research.143730.r200814

**Published:** 2023-11-27

**Authors:** Nawab John Dar

**Affiliations:** 1 School of Medicine, The University of Texas Health Science Center, San Antonio, Texas, USA

The study entitled " Effects of Ashwagandha ( *Withania somnifera*) standardized root extract on physical endurance and VO _2max_ in healthy adults preforming resistance training: An eight-week, prospective, randomized, double-blind, placebo-controlled study by Verma *et al*, is well designed and conducted, however, I have few queries before it is approved for indexing:

1. The authors did not mention about the habits of the volunteers, what about their smoking habits and sleep cycle?
2. Who manufactured the AG capsules and how did they made sure Quality of the capsules (QC&QA), why would they buy the control and AG from different companies?
3. I would love to know what happened to the endurance once the study is over and the volunteers stopped taking AG? 

Is the work clearly and accurately presented and does it cite the current literature?

Yes

If applicable, is the statistical analysis and its interpretation appropriate?

Partly

Are all the source data underlying the results available to ensure full reproducibility?

Yes

Is the study design appropriate and is the work technically sound?

Yes

Are the conclusions drawn adequately supported by the results?

Partly

Are sufficient details of methods and analysis provided to allow replication by others?

Yes

Reviewer Expertise:

Neuropharmacology

I confirm that I have read this submission and believe that I have an appropriate level of expertise to confirm that it is of an acceptable scientific standard, however I have significant reservations, as outlined above.

## References

[1] : Pharmacologic overview of Withania somnifera, the Indian Ginseng. *Cell Mol Life Sci* .2015;72(23) : 10.1007/s00018-015-2012-1 4445-60 10.1007/s00018-015-2012-1 26306935 PMC11113996

# Response

**Published:** 2024-04-02

**Authors:** sayali patil

Dear Nawab John Dar,

 Thank you for your time and valuable feedback on our manuscript. We have carefully reviewed your comments. As suggested, we provide a point-by-point response as below:

- *The authors did not mention about the habits of the volunteers, what about their smoking habits and sleep cycle?*

**The two groups were similar with respect to the personal history and habits. A statement to that effect is added in the “subjects” section of the article (version 2).**

  

- *Who manufactured the AG capsules and how did they made sure Quality of the capsules (QC&QA), why would they buy the control and AG from different companies?*

**Control capsules were manufactured specifically for the study purpose and hence, it was outsourced to third party manufacturing. However, the study capsules (AG) were provided to manufacturer to match (identical placebo) the control (PB) capsules with AG.**

  

- *I would love to know what happened to the endurance once the study is over and the volunteers stopped taking AG?*

**Follow-up was not a part of the study plan and we did not assess this.**

# Reviewer report: Reviewer response for version 1

**DOI:** 10.5256/f1000research.143730.r219543

**Published:** 2023-11-13

**Authors:** Mark E. T. Willems

**Affiliations:** 1 Institute of Applied Sciences, College Lane, University of Chichester, Chichester, England, UK

In the title, change “preforming”.

 Please clarify In the background section of the abstract that 600 mg was used.

 Please revise in the background of the abstract that muscle mass was not measured. An indirect measurement of cross sectional area seems to have been measured.

 Please have specific conclusions. There are no measurements of recovery. Please revise the conclusion in the abstract.

 The introduction does not provide justification for the need to examine the effects in females. Please provide that justification.

 P3, Does we need the statement supported by reference 6. It is very misleading and has nothing to do with the study.

 P3. Just linking physical endurance in males with testosterone is an extreme simplification of all the factors and determinants of physical endurance. Please revise.

 There is repeat in the method section regarding ethical approval. Please revise.

 The methods indicate that participants could take the capsules with milk. Please provide the number of participants in each group that consumed the capsules with milk. Milk intake may enhance protein synthesis and may have affected some of the parameters of interest. This needs to be discussed and probably acknowledged as a limitation of the study.

 The sample size calculation is confusing. It seems 40 participants were required in each group and with consideration of 10% drop-out, 44-45 participants should have been in each group. Please clarify.

 On P5 is mentioned “standard biochemistry tests”. Please provide more information.

 Table 1. I suggest to provide values without decimal places.

 Table 2. Do you need to express values with two decimal places? Please reconsider.

 Please be consistent with terminology. Is the bench press the same as the chest press?

 Table 3. Are you allowed to express the differences with three decimal places? For example, with circumference changes expressed with 3 decimal places indicates a measurement to the nearest 0.001 cm. I am sure that was not done.

 The p-values in the text in the result section does not match with the information provided in the Tables. For example “There were significant differences between the groups in both 1 RM bench press (males: effect size, 0.91; p=0.0084; females: effect size, 1.56; p=0.0005; total: effect size, 1.14; p<0.0001)”. Please ensure that text information matches table information.

 Figs 3 and 4 has data that is communicated as well in Table 3. Please avoid duplication. I suggest to delete Figs 3 and 4 as it does not present new information.

 P10. Please provide the outcome of the clinical tests.

Is the work clearly and accurately presented and does it cite the current literature?

Yes

If applicable, is the statistical analysis and its interpretation appropriate?

Yes

Are all the source data underlying the results available to ensure full reproducibility?

Yes

Is the study design appropriate and is the work technically sound?

No

Are the conclusions drawn adequately supported by the results?

No

Are sufficient details of methods and analysis provided to allow replication by others?

No

Reviewer Expertise:

Exercise Physiology, Training, Sports Nutrition

I confirm that I have read this submission and believe that I have an appropriate level of expertise to state that I do not consider it to be of an acceptable scientific standard, for reasons outlined above.

# Response

**Published:** 2024-04-02

**Authors:** sayali patil

Dear Mark E. T. Willems,

 Thank you for your time and valuable feedback on our manuscript. We have carefully reviewed your comments. As suggested, we provide a point-by-point response as below:

- *In the title, change “preforming”.*

**Correction from “preforming” to “performing” in the title has been made for version 2 of the article.**

  

- *Please clarify in the background section of the abstract that 600 mg was used.*

**Correction from 300mg to 600mg of Ashwagandha has been made for version 2 of the article. [page 1, paragraph 1]**

  

- *Please revise in the background of the abstract that muscle mass was not measured. An indirect measurement of cross-sectional area seems to have been measured.*

**As suggested “muscle mass” in abstract is replaced with “muscle size” in the version-2 of the article. (page 1, paragraph 1; page 3, paragraph 3; page 4, paragraph 6)**

  

- *Please have specific conclusions. There are no measurements of recovery. Please revise the conclusion in the abstract.*

**We acknowledge that there were no measurements of recovery. Revision has been made for version 2 of the article. [page 2, paragraph 2]**

  

- *The introduction does not provide justification for the need to examine the effects in females. Please provide that justification.*

**“Since gender is an important factor affecting serum hormone levels (and subsequent response to effects of exercise) due to physiological factors, this study focussed on testing the responses in both the genders who are regularly active exercising. There is scarcity of data in previously published literature.”**

**This above statement has been added in the paragraph 5 on page 3.**

  

- *P3, do we need the statement supported by reference 6. It is very misleading and has nothing to do with the study.*

**“Biochemical assessments reveal that individuals who regularly participate in endurance exercise have significantly higher creatine kinase levels, which peak approximately 48 hours after the exercise.”**

**This statement on page-3 is rephrased to improve clarity as below:**

**“Serum creatine kinase is raised in individuals who regularly participate in endurance exercise, with peak levels seen 48 hours after the exercise.”**

  

- *P3. Just linking physical endurance in males with testosterone is an extreme simplification of all the factors and determinants of physical endurance. Please revise.*

**“A previously published study evaluating muscle strength focused only on male participants. ^18^ Physical endurance is considered higher in males owing it to the testosterone hormone.”**

**The sentences are revised as below:**

**“A previously published study evaluating muscle strength focused only on male participants. ^18^ However, data on muscle strength and endurance in female participants is scarce.”**

  

- *There is repeat in the method section regarding ethical approval. Please revise.*

**We have removed the repetition and ethical approval details are mentioned only in the “ethical approval” section under Methods.**

  

- *The methods indicate that participants could take the capsules with milk. Please provide the number of participants in each group that consumed the capsules with milk. Milk intake may enhance protein synthesis and may have affected some of the parameters of interest. This needs to be discussed and probably acknowledged as a limitation of the study.*

**Two males and one female consumed capsules with milk in AG group, whereas 3 males and one female in PB group consumed capsules with milk.**

**This information has been included in the “Interventions” section of the article (version 2). The same has been included as a limitation of the study as suggested.**

  

- *The sample size calculation is confusing. It seems 40 participants were required in each group and with consideration of 10% drop-out, 44-45 participants should have been in each group. Please clarify.*

**Since it was expected that the study participants were highly motivated adults performing regular exercises, we had not anticipated data loss due to lost to follow-up. However, we had data loss not due to lost to follow-up, but due to poor compliance (3 participants in AG and 4 in PB group). We failed to anticipate this possibility during study planning. However, we estimated the effect size (1.14 and 1.11 for 1-RM bench press and 1-RM leg press respectively) for the primary outcomes, to derive our conclusions.**

  

- *On P5 is mentioned “standard biochemistry tests”. Please provide more information.*

**Please find the outcomes of the “standard biochemistry tests” as clinical tests (hematology, renal function, liver function and thyroid function tests) included in the article’s Data Availability section as “Extended data”.**

  

- *Table 1. I suggest to provide values without decimal places.*

**All 3 tables in the article uniformly have values with a single decimal place in the version-2 of this article.**

  

- *Table 2. Do you need to express values with two decimal places? Please reconsider.*

**All 3 tables in the article uniformly have values with a single decimal place in the version-2 of this article.**

  

- *Please be consistent with terminology. Is the bench press the same as the chest press?*

**We have made changes and kept the terminology as “bench press” throughout the article in the version 2.**

  

- *Table 3. Are you allowed to express the differences with three decimal places? For example, with circumference changes expressed with 3 decimal places indicates a measurement to the nearest 0.001 cm. I am sure that was not done.*

**Thank you for the observation. All 3 tables in the article uniformly have values with a single decimal place in the version-2 of this article.**

  

1. *The p-values in the text in the result section does not match with the information provided in the Tables. For example, “There were significant differences between the groups in both 1 RM bench press (males: effect size, 0.91; p=0.0084; females: effect size, 1.56; p=0.0005; total: effect size, 1.14; p<0.0001)”. Please ensure that text information matches table information.*

**Kindly note that in the result section (page 8, paragraph 1), p-values in the text are with respect to the effect size whereas p-values in tables are with respect to the comparison of means (t-test).**

  

- *Figs 3 and 4 has data that is communicated as well in Table 3. Please avoid duplication. I suggest to delete Figs 3 and 4 as it does not present new information.*

**Thank you for the observation. We have removed Figures 3 and 4 for the version 2 of the article.**

  

- *P10. Please provide the outcome of the clinical tests.*

**Please find the outcomes of the clinical tests (hematology, renal function, liver function and thyroid function tests) included in the article’s Data Availability section as “Extended data”.**
